# Influence of Broad-Spectrum Mycotoxin Detoxifiers on Growth, Jejunal Morphology, Liver Histopathology and Oxidative Stress in Broilers Fed Diets Contaminated with Multiple Mycotoxins

**DOI:** 10.3390/vetsci13040362

**Published:** 2026-04-08

**Authors:** Orawan Suthtirak, Thaweesak Songserm, Koonphol Pongmanee, Kazeem D. Adeyemi, Konkawat Rassmidatta, Ricardo Communod, Yemi Burden, Damien P. Preveraud, Yuwares Ruangpanit

**Affiliations:** 1 Department of Animal Science, Faculty of Agriculture at Kamphaeng Saen, Kasetsart University, Kamphaeng Saen Campus, Nakhon Pathom 73140, Thailand; orawan.suth@ku.th (O.S.); koonphol.p@ku.th (K.P.); konkawat.ras@ku.th (K.R.); 2 Department of Pathology, Faculty of Veterinary Medicine, Kasetsart University, Bangkok 10900, Thailand; fvettss@nontri.ku.ac.th; 3 Department of Animal Production, Faculty of Agriculture, University of Ilorin, Ilorin 240003, Nigeria; adeyemi.kd@unilorin.edu.ng; 4 Adisseo Asia Pacific Private Limited, Singapore 048581, Singapore; ricardo.communod@adisseo.com; 5 Adisseo ELISE (European Labs for Innovation, Science, and Expertise), 69190 Saint Fons, France; yemi.burden@adisseo.com; 6 Adisseo France Société par Actions Simplifiée, 92160 Antony, France

**Keywords:** crypt depth, fatty liver, interleukin-10, liver lesion, villus height

## Abstract

The effects of broad-spectrum mycotoxin detoxifiers (BSMDs) on growth performance, liver histopathology, jejunal morphology, and oxidative stress were evaluated in broilers fed diets contaminated with multiple mycotoxins. A total of 800 one-day-old male Ross 308 broilers were randomly assigned to four treatments, with eight replicates of 25 birds each, and reared for 42 days. The treatments included a basal control diet (CON); a multi-mycotoxin-contaminated diet (MMT) containing aflatoxins (25 µg/kg), zearalenone (135 µg/kg), T2 toxin (85 µg/kg), fumonisin (1.90 mg/kg), and deoxynivalenol (0.70 mg/kg); and the MMT diet supplemented with either 1.0 kg/ton BSMD-1 or 1.5 kg/ton BSMD-2. MMT contamination did not affect growth performance, serum malondialdehyde, interleukin-6 levels, liver enzyme activities, or liver lesion scores. Nevertheless, interleukin-10 levels were lower in birds fed the MMT diet (*p* = 0.03). In birds fed MMT, there was a substantial decrease (*p* < 0.05) in the height of the jejunal villi, their surface area, and the ratio of their height to the depth of the crypt. While BSMD-supplemented groups displayed values similar to both CON and MMT, MMT birds had higher fatty liver scores than the control group. Overall, multi-mycotoxin contamination impaired gut morphology and immune balance. BSMD supplementation improved intestinal structure, enhanced immune response, and partially mitigated liver alterations. These findings indicate its potential as a dietary intervention to mitigate the detrimental effects of multi-mycotoxin contamination in broilers.

## Simple Summary

This study investigated whether broad-spectrum mycotoxin detoxifiers (BSMDs) can protect broiler chickens from the harmful effects of multiple mycotoxins in feed. Although the contaminated diet did not reduce growth or increase oxidative stress or liver enzyme levels, it weakened immune regulation, damaged the gut lining, and negatively affected liver health. Adding BSMD to the contaminated diet improved gut structure, supported immune function, and partially reduced liver damage. These findings show that even when growth appears normal, hidden health problems can occur under multi-mycotoxin exposure, and detoxifiers may help reduce these risks.

## 1. Introduction

Mycotoxin contamination is a persistent challenge in poultry production and remains a major threat to animal health, growth, and product quality [1,2]. These toxic secondary metabolites are produced mainly by Aspergillus, Fusarium, and Penicillium species that often colonize cereal grains used for poultry feed [3,4,5]. In tropical climates, where humidity and temperature enhance fungal growth, contamination is common and difficult to avoid [6,7]. In poultry production, aflatoxins, zearalenone, T-2 toxin, deoxynivalenol, and fumonisins are widely recognized as the primary mycotoxins of nutritional and health significance [8,9]. These toxins frequently co-occur in feed ingredients, where their combined effects may exceed those of individual toxins, even at apparently low levels [7,10,11].

Aflatoxins impair liver function and suppress immunity [7,12]. Zearalenone alters metabolic and reproductive processes and can disrupt intestinal physiology [13,14]. T2 toxin is strongly cytotoxic and damages the intestinal epithelium [15,16]. Deoxynivalenol compromises gut barrier integrity and promotes oxidative stress [14,17,18]. Fumonisins interfere with sphingolipid metabolism and cause intestinal and hepatic lesions [7,19]. When these toxins are present simultaneously, they can reduce feed intake, depress growth performance, damage the intestine, and stimulate oxidative reactions, which ultimately decrease productivity in broiler productions [7,20].

Since it is not possible to completely remove mycotoxins from feed ingredients, multi-component mycotoxin detoxifiers have emerged as a crucial management strategy [21,22,23,24]. In order to work through complementary mechanisms such as gastrointestinal adsorption of toxins, support of endogenous detoxification and tissue repair pathways, and mitigation of mycotoxin-induced oxidative stress, these detoxifiers usually combine clay minerals, yeast derivatives, organic acids, antioxidants, and botanical extracts [25,26,27].

The gastrointestinal tract is the first site of exposure to dietary mycotoxins and is particularly vulnerable to their toxic effects [28,29,30]. Intestinal damage, characterized by a shorter villi and deeper crypts, reduces the functional absorptive surface and impairs nutrient uptake, ultimately compromising growth performance [17,28,30]. Beyond structural injury, many mycotoxins activate oxidative pathways, leading to increased oxidative stress and suppression of antioxidant enzyme activity [31,32,33]. The liver, as the principal organ involved in detoxification and metabolism, is similarly vulnerable to mycotoxin challenge, with chronic exposure commonly resulting in hepatic steatosis, inflammatory responses, and oxidative injury [34,35,36,37]. Together, these intestinal and hepatic impairments adversely affect nutrient utilization and overall performance. Consequently, evaluation of intestinal morphology, oxidative stress biomarkers, and liver histopathology represents a relevant and integrated approach for assessing the mycotoxin toxicity and the efficacy of dietary detoxification strategies in broilers.

Although a lot of study has been done on individual toxins, there aren’t many studies assessing broad-spectrum mycotoxin detoxifiers (BSMD) under multi-mycotoxin contamination. This is a serious problem for commercial broiler production. Furthermore, there aren’t many comparative evaluations of various detoxifier compositions under these circumstances. Therefore, the key research question of this study was whether BSMD supplementation can effectively mitigate the detrimental effects of multi-mycotoxin contamination in broiler diets. We hypothesized that dietary inclusion of BSMDs would alleviate mycotoxin-induced impairments by improving growth performance, preserving liver health and intestinal morphology, reducing inflammation, and enhancing antioxidant status. Accordingly, this study aimed to evaluate the effects of BSMDs on growth performance, jejunal morphology, liver histopathology, and oxidative stress in broilers exposed to multiple mycotoxins.

## 2. Materials and Methods

### 2.1. Biosafety Measures During Experimental Procedures

In this experiment, all personnel were required to wear complete personal protective equipment (PPE) throughout procedures involving feed or materials potentially contaminated with aflatoxins, in accordance with standard laboratory practices that classify mycotoxins as biohazardous substances. The PPE included nitrile gloves to prevent direct dermal contact; safety goggles or face shields to protect against airborne particles or splashes; and disposable masks or N95 particulate respirators to reduce inhalation of contaminated dust generated during the handling or sorting of feed materials. In addition, personnel wore laboratory coats, gowns, or disposable protective suits over personal clothing, as well as impermeable footwear or shoe covers, to minimize cross-contamination between work areas. All PPE was removed prior to leaving the work area, followed by thorough handwashing. All procedures were conducted in adequately ventilated environments to further reduce exposure risks.

Waste generated during the experiment including leftover feed, residual materials, and excreta suspected of aflatoxin contamination, was collected in designated, tightly sealed containers clearly labeled as hazardous waste and disposed of through the institution’s certified hazardous waste management system. At the conclusion of the experiment, broiler carcasses were treated as biological waste and disposed of through controlled burial within authorized systems or designated areas. All procedures were conducted in accordance with internationally recognized biosafety and occupational health guidelines to minimize the risks associated with aflatoxin exposure to humans, animals, and the environment [38,39,40,41].

### 2.2. Mycotoxins Preparation and Analysis

The mycotoxins were produced by isolates of *Aspergillus flavus* (aflatoxin), *Fusarium graminearum* (deoxynivalenol and zearalenone), *Fusarium verticilloides* (fumonisin), and *Fusarium sporotrichioides* (T-2 toxin). Fungal isolates were obtained from grains collected from production fields. These isolates were used to ferment parboiled rice for the production of aflatoxin (AF), deoxynivalenol (DON), and zearalenone (ZEA), or corn for fumonisin (FUM) and T-2 toxin, following the protocol described by Torteli et al. [42]. Briefly, 100 g of rice or corn was transferred into 500 mL Erlenmeyer flasks, and 40 mL of distilled water was added at least 2 h prior to sterilization and thoroughly mixed with the substrate. The flasks were plugged with cotton and sterilized at 121 °C for 30 min, then allowed to cool to room temperature before inoculation. The sterilized substrates were inoculated with 2 mL of a spore suspension (10^8^ spores/mL) prepared from fungal cultures grown on potato dextrose agar for 7 days. Incubation was carried out under static conditions at 25 °C in the dark for 21 days. After incubation, the fermented material was dried in an oven at 57 °C and ground using a mill fitted with a sieve (mesh size < 0.85 mm). All mycotoxins were prepared at the Laboratory of the Biology Department, ESALQ, University of São Paulo, Brazil. The resulting fermented material contained 0.196 µg/kg AFs, 0.05 µg/kg ZEA, 0.243 µg/kg T-2 toxin, 256 mg/kg FUM, and 105.3 mg/kg DON. Mycotoxin concentrations were determined using enzyme-linked immunosorbent assay (ELISA) with commercial kits: DON (Veratox^®^ DON 2/3 Quantitative Test Kit (Neogen, MI, USA), 0.5–6.0 ppm), ZEA (AgraQuant^®^ Zearalenone Plus Test Kit (Romer Labs Singapore Pte. Ltd, Singapore), 25–1000 ppb), T-2 toxin (Veratox^®^ for T-2/HT-2 (Neogen, MI, USA), 25–250 ppb), fumonisin (AgraQuant^®^ Fumonisin ELISA Test Kit (Romer Labs Singapore Pte. Ltd, Singapore), 0.25–5.0 ppm), and total aflatoxins (AgraQuant^®^ Aflatoxin ELISA Test Kit (Romer Labs Singapore Pte. Ltd, Singapore), 4–40 ppb). The analyzed contents of mycotoxins in the experimental diets are presented in Table 1.

### 2.3. Birds, Management, Diets, and Experimental Design

A total of 800 one-day-old male Ross 308 broilers were randomly allocated to four dietary treatments, each with eight replicates of 25 birds, and reared for 42 days. The treatments were: a basal diet (control; CON); the basal diet contaminated with AFs (25 µg/kg), ZEA (135 µg/kg), T2 toxin (85 µg/kg), FUM (1.90 mg/kg), and DON (0.70 mg/kg) (MMT); MMT plus 1.0 kg/ton BSMD-1 (UNIKE^®^ Plus, Adisseo, Antony, France; composed of bentonite, sepiolite, inactivated yeast and yeast extracts from *Saccharomyces cerevisiae*, and botanicals); and MMT plus 1.5 kg/ton BSMD-2 (TOXY-NIL^®^ Plus, Adisseo, Antony, France; containing bentonite, sepiolite, inactivated yeast and yeast extracts, and botanicals).

The birds were housed in an evaporative cooling facility on concrete floors with rice husk litter. Feed (pelleted) and water were provided *ad libitum* throughout the experiment. The lighting regimen included 23 h of light and 1 h of darkness for the first 7 days and 20 h of light and 4 h of darkness from 8–42 days. The vaccination schedule consisted of Newcastle disease (ND; B1 strain) and infectious bronchitis (IB) on day 7 via nasal drops; infectious bursal disease (IBD) on day 14 via oral drops; and ND (LaSota strain) plus IB on day 21 via nasal drops.

All experimental diets were based on corn and soybean meal and formulated according to Ross 308 nutritional recommendations. The study consisted of three feeding phases over 42 days. Ingredient composition and calculated nutrient levels of the diets are presented in Table 2 and Table 3. The proximate composition of the experimental diets was determined according to standard methods of AOAC International [43], including moisture (Method 934.01), crude protein (Method 984.13), ether extract (Method 920.39), ash (Method 942.05), and crude fiber (Method 978.10), and the results are presented in Table 4.

### 2.4. Growth Performance and Antibody Titer Analysis

Body weight (BW) and feed intake (FI) were recorded on days 1, 14, 21, 35, and 42 for the calculation of body weight gain (BWG) and feed conversion ratio (FCR) during the trial. Daily temperature and relative humidity were also recorded. On d 24, two broilers per replicate were randomly selected for blood collection to assess Newcastle disease (ND) and infectious bronchitis (IB) antibody titers. ND and IB titers were determined using the hemagglutination inhibition (HI) assay for Newcastle disease virus (NDV) and infectious bronchitis virus (IBV), respectively. HI antibody values were then expressed as log_2_ titers (log_2_ transformation).

### 2.5. Analysis of Serum Parameters and Liver Enzymes Activity

On d 42, two broilers per replicate were randomly selected for blood collection. Blood samples were collected from the wing vein using plain vacutainer tubes and were left to clot at room temperature. Thereafter, the samples were centrifuged at 3000× *g* for 10 min to separate the serum and stored at −20 °C until analysis. Serum was analyzed for biomarkers of oxidative stress, glutathione peroxidase (GSH-PX) and malondialdehyde (MDA), and for inflammatory biomarkers, including interleukin-6 (IL-6) and interleukin-10 (IL-10). Serum GSH-PX, IL-6, and IL-10 concentrations were quantified using commercially available chicken-specific ELISA kits (GSH-PX: MBS7608110; IL-6: MBS288783; IL-10: MBS701683; MyBioSource Inc., San Diego, CA, USA), following the manufacturer’s instructions. Serum MDA levels were determined using a modified thiobarbituric acid reactive substance (TBARS) assay as described by Grotto et al. [44]. In addition, the activities of aspartate aminotransferase (AST), alanine aminotransferase (ALT), and gamma-glutamyl transferase (GGT) were measured using an automatic clinical chemistry analyzer (ILab Aries, Instrumentation Laboratory SpA, Milan, Italy) to assess hepatic function.

### 2.6. Analysis of Jejunal Morphology

On d 42, two broilers per replicate were randomly selected and euthanized by CO_2_ inhalation [45]. Jejunal samples were collected from the mid-point of the jejunum and processed for histological examination following a modified method described by Iji et al. [46]. Briefly, a 2-cm intestinal segment was fixed in 10% buffered formalin, embedded in paraffin, sectioned at 5-µm thickness, and stained with hematoxylin and eosin. Villus height (VH), villus width (VW), and crypt depth (CD) were measured under 40× magnification using an Olympus DP22 digital camera and the DP2-SAL image analysis system (Olympus Optical Corp., Tokyo, Japan). The VH-to-CD ratio (VH:CD ratio) was then calculated. Villus surface area (VSA) was estimated using the following formula: (2π) × (half of VW) × VH, as described by Sakamoto et al. [47].

### 2.7. Fatty Liver and Liver Histopathology Analysis

The severity of fatty liver was assessed on day 42 by euthanizing two birds per pen, excising the liver, and evaluating its coloration, pathological lesions, histological features, and macroscopic alterations (including inflammation and hemorrhage), following the procedures described by Choi et al. [48]. Histological slides were examined under 400× magnification using an optical microscope, according to Malisorn et al. [49]. The fatty liver scores were defined as follows:

Score 1, normal liver with a dark red color; Score 2, mild fatty liver hemorrhagic syndrome (FLHS), characterized by a slightly yellow liver and mild hemorrhages; Score 3, moderate FLHS, presenting as a light yellowish-red liver with moderate hemorrhages; Score 4, severe FLHS, characterized by an enlarged, putty-colored liver with large or massive hemorrhages.

The liver lesion scores were defined as follows: Score 0, normal liver; Score 1, mild fatty degeneration, characterized by vacuolar (hydropic) degeneration of hepatic cells affecting < 50% of the tissue area; Score 2, extensive vacuolar degeneration throughout the hepatic parenchyma with occasional foci of hepatitis; Score 3, severe vacuolar degeneration of the hepatic parenchyma with moderate to severe hepatitis, characterized by infiltration of inflammatory cells into the parenchyma; and Score 4, severe, extensive hepatitis with fibrosis and features suggestive of possible cirrhosis, including bile duct proliferation and hyperplasia.

### 2.8. Statistical Analysis

Data obtained were assessed for normality using the Shapiro–Wilk test and for homogeneity of variance using Levene’s test before statistical analysis. All data, including growth performance, serum biochemical parameters, and liver enzyme activities, except liver fatty and lesion scores, were subjected to analysis of variance appropriate for a completely randomized design using the GLM procedure of SAS (version 9.4; SAS Institute Inc., Cary, NC, USA). Duncan’s multiple range test was used to separate treatment means at *p* < 0.05. Liver fatty and lesion scores were analyzed using the Kruskal–Wallis test.

## 3. Results

### 3.1. Growth Performance and Antibody Titer

Contamination with MMT did not affect BWG, FI, or FCR in broilers throughout the 42-day period (Table 5). Similarly, hemagglutination inhibition titers for ND and IB on d 24 were not influenced by dietary treatments (Table 6).

### 3.2. Serum Parameters and Liver Enzymes Activity

On d 42, serum GSH-PX activity, MDA concentration, and IL-6 levels and the activities of AST, ALT, and GGT were not affected (*p* > 0.05) by the dietary treatments (Table 7). However, serum IL-10 concentration was lower in the MMT group than in the other dietary groups (*p* = 0.015).

### 3.3. Jejunal Morphology

Birds in the MMT group had reduced (*p* < 0.05) villus height, villus height-to-crypt depth ratio, and jejunal surface area compared with the other dietary groups (Table 8). In contrast, villus width and crypt depth were not affected by diet (*p* > 0.05).

### 3.4. Fatty Liver and Liver Histopathology

Fatty liver score was higher (*p* = 0.03) in the MMT group than in the CON group (Table 9). Fatty liver scores of birds fed BSMD-1 or BSMD-2 did not differ from those of either CON or MMT birds. Liver lesion scores were not affected by dietary treatments (Table 10).

## 4. Discussion

Multi-mycotoxin contamination is a widespread problem in poultry production, particularly in tropical regions, and can negatively affect growth performance, gut health, liver function, and oxidative balance in broilers. While several studies have examined the effects of individual mycotoxins, birds are often exposed to multiple toxins simultaneously, which can have additive or synergistic harmful effects. Broad-spectrum mycotoxin detoxifiers have been proposed as a dietary strategy to mitigate these effects through adsorption, support of detoxification pathways, and antioxidant protection. In this study, we evaluated whether dietary inclusion of BSMD could protect broilers from the adverse effects of multi-mycotoxin exposure, focusing on growth performance, intestinal morphology, liver histopathology, and oxidative stress.

Growth metrics of broilers were not affected by diets contaminated with multiple mycotoxins. This suggests that the concentrations of aflatoxins, T-2 toxin, fumonisins, and deoxynivalenol were insufficient to elicit overt performance impairment typically associated with mycotoxicosis. Furthermore, the levels of all mycotoxins in the experimental diets were maintained below regulatory thresholds. Aflatoxin levels were lower than the maximum limit set by the Department of Livestock Development, Thailand [50], while fumonisin, deoxynivalenol, T-2 toxin, and zearalenone levels were below the guidance values established by the European Commission [51].

Although growth performance was not affected by dietary exposure to multiple mycotoxins, these results should be interpreted in the context of the experimental setup. Typically, commercial broiler systems are inundated with combined exposure to resident pathogen pressure and environmental stress, which may exacerbate the physiological consequences of low-level mycotoxin exposure, potentially resulting in measurable performance responses. The present study was conducted under controlled research conditions that minimize background stressors.

Consistent with the present findings, dietary contamination with 0.05 ppm aflatoxin and 20 ppm fumonisin did not affect body weight gain or feed conversion ratio from day 1 to 21, nor feed intake from day 1 to 35 in broilers [34]. Likewise, inclusion of 5 ppm DON in broiler diets did not impair growth performance [35,52]. Similarly, subclinical levels of deoxynivalenol, zearalenone, and fumonisins in broiler diets did not influence growth metrics [53].

The antibody titers against infectious bronchitis and Newcastle disease were unaffected by multiple mycotoxin contamination, indicating that the broiler immune system was still functionally intact during the trial. Since humoral immunity is typically only seen at larger or longer mycotoxin exposures, the toxin concentrations employed were probably below the threshold needed to inhibit it. Trichothecenes, fumonisins, aflatoxins, and deoxynivalenol primarily cause moderate oxidative or inflammatory reactions at subclinical levels, but these effects are frequently insufficient to impair B-cell function, antibody production, or vaccine responsiveness [53].

Serum GSH-PX activity, MDA, IL-6, AST, ALT, and GGT levels were not significantly changed by multiple mycotoxin contamination, suggesting that the toxin concentrations employed were insufficient to cause detectable oxidative stress, systemic inflammation, or hepatic injury in broilers. Increased lipid peroxidation, elevated liver enzymes, and impaired antioxidant defenses are generally the results of larger or longer exposures to aflatoxins, trichothecenes, fumonisins, and DON [54,55]. Nonetheless, the antioxidant system and hepatic detoxification processes of birds are probably capable of efficiently metabolizing the poisons at the subclinical levels found in our work, preserving stable biochemical markers.

Although most parameters remained unaffected, mixed mycotoxin contamination caused a reduction in serum IL-10, an anti-inflammatory cytokine that helps regulate immune homeostasis. This reduction suggests that even at relatively low concentrations, the combined toxins may exert subtle immunomodulatory effects that skew cytokine balance toward a less anti-inflammatory state. Such an effect is consistent with the ability of certain mycotoxins, particularly trichothecenes, to disrupt cytokine signaling in immune cells even when overt physiological damage is not evident [56]. This finding aligns with Qing et al. [57], in which feeding diet contaminated with 0.101 ppm Ochratoxin A + 0.02 ppm AFB1 lowered IL-10 in pullets. Interestingly, broilers receiving both the multiple mycotoxins and BSMDs exhibited IL-10 concentrations comparable to the uncontaminated control group. This indicates that the detoxifiers effectively reduced the bioavailability or intestinal absorption of the toxins, thereby preventing their immunomodulatory effects on IL-10 synthesis. By adsorbing or inactivating the toxins in the gut, the detoxifiers likely protected immune cells from toxin-induced signaling disruption.

Multiple mycotoxin contamination significantly reduced villus height, villus surface area, and the villus height-to-crypt depth ratio, indicating impaired intestinal morphology and absorptive capacity. Mycotoxins such as aflatoxins, T-2 toxin, DON, and FUM are known to damage enterocytes, inhibit protein synthesis, and induce local inflammation within the gut [7,14,19]. These effects collectively lead to villus atrophy and crypt hyperplasia, reducing the functional epithelial surface available for nutrient absorption [17,58]. The decline in VH:CD ratio further reflects a shift toward increased crypt cell turnover, a compensatory response to epithelial injury. These results align with reports from Cheng et al. [59] who observed reductions in villus height and VH:CD ratio in broilers fed diet contaminated with 11 ppm AFB1 + 0.4 ppm ZEA +1.8 ppm DON for 42 d. In addition, the contamination of diet with 14–33 ppm FUM + 1–3.5 ppm DON + 0.2–0.8 ppm ZEA lowered ileal and jejunal villus height in broilers [53].

However, broilers receiving both mixed mycotoxins and BSMDs exhibited villus height, villus surface area, and VH:CD ratios comparable to those of birds fed uncontaminated diets. This suggests that the detoxifiers were effective in limiting intestinal exposure to the toxins, likely by adsorbing toxin molecules in the lumen and preventing their interaction with the gut epithelium. By reducing toxin absorption and epithelial irritation, the detoxifiers helped maintain normal villus architecture and epithelial renewal dynamics. Similarly, the supplementation of mycotoxin binders to mycotoxin-contaminated diets led to the restoration of gut morphology in broilers [35,59,60].

Mixed mycotoxin contamination increased the fatty liver score in broilers, indicating that the combined toxins exerted measurable effects on hepatic lipid metabolism and structural integrity. Mycotoxins can impair hepatic function by interfering with lipid transport, inhibiting protein synthesis, and inducing oxidative stress in hepatocytes [61]. These disruptions can lead to lipid accumulation, mild hemorrhaging, and discoloration of the liver, features reflected in higher fatty liver scores (scores 2–3), which denote early to moderate fatty liver hemorrhagic syndrome [57,62].

Interestingly, broilers fed diets containing both multiple mycotoxins and BSMD exhibited fatty liver scores comparable to those fed uncontaminated diets, as well as those fed mixed mycotoxins alone. This suggests two important patterns. First, although mycotoxins elevated the fatty liver score relative to a normal liver (score 1), the severity remained within a mild to moderate range and did not progress to severe pathological levels. Second, the lack of difference between the BSMD-treated groups and both CON and MMT groups implies that the detoxifiers offered some level of protection.

Multiple mycotoxin contamination did not influence liver lesion scores in broilers, indicating that the toxin levels used in the study were insufficient to produce overt hepatic pathology detectable by gross examination. Although mycotoxins such as aflatoxins, DON, fumonisins, and T-2 toxins can induce hepatocellular damage, oxidative stress, and vascular changes; these effects typically become evident at higher concentrations or with longer exposure periods. At the subclinical levels present in this experiment, the liver’s detoxification capacity, through phase I and II enzymes, antioxidant defenses, and regenerative ability, likely mitigated potential damage before it progressed to visible lesions. The absence of significant lesions also aligns with the stable serum liver enzyme activities (AST, ALT, GGT), which collectively suggests that hepatocellular integrity was largely preserved. Consistently, feeding diets contaminated with Aflatoxin-B1 and Ochratoxin did not affect liver lesion score in broilers [63]. Overall, the findings demonstrate that multiple mycotoxins at the tested concentrations did not induce gross hepatic injury, reflecting effective hepatic resilience or detoxification under the conditions of the study.

This study has some limitations. First, it was conducted exclusively in broilers, which may limit the direct extrapolation of the findings to other poultry species. Second, while broad-spectrum mycotoxin detoxifiers may influence the gut microbiome, this aspect was not investigated and warrants further research. Third, the detailed molecular mechanisms underlying mycotoxin toxicity were not explored, as the study focused on growth performance, intestinal morphology, liver histopathology, and oxidative stress. Finally, measurements of mycotoxin residues in tissues or blood were not included, which could provide direct evidence of detoxifier efficacy in vivo. Future studies addressing these gaps will provide a more comprehensive understanding of the protective effects of mycotoxin detoxifiers in poultry production.

## 5. Conclusions

Dietary contamination with multiple mycotoxins in broilers did not significantly affect growth performance, serum malondialdehyde, antioxidant enzyme activity, liver enzyme levels, or antibody titers against Newcastle disease and infectious bronchitis. However, it caused a significant reduction in IL-10 levels, indicating compromised anti-inflammatory and immune-regulatory functions. Moreover, multiple mycotoxin exposure disrupted jejunal morphology, as evidenced by reduced villus height, villus height-to-crypt depth ratio, and absorptive surface area, and worsened fatty liver condition, as reflected by higher fatty liver scores compared to control birds. Supplementation with broad-spectrum mycotoxin detoxifiers (BSMD-1 and BSMD-2) effectively alleviated the adverse effects on jejunal structure and immune regulation and partially mitigated the impact on fatty liver. These findings suggest that while moderate mycotoxin contamination may not overtly affect growth or serum biochemical markers, it can compromise gut integrity and immune homeostasis. The use of BSMD represents a practical strategy to enhance resilience against naturally occurring mixed mycotoxins in commercial broiler production, improving intestinal health, immune function, and overall welfare.

## Figures and Tables

**Table 1:** Analyzed levels of mycotoxins in experimental diets.

Mycotoxin	Dietary Treatments ^1^			
	CON	MMT	BSMD-1	BSMD-2
Starter (1–14 d)				
Aflatoxins (µg/kg)	5.1	33.4	25.4	27.4
Zearalenone (µg/kg)	<25.0	137.9	142.6	173.4
T-2 toxin (µg/kg)	<25.0	97.7	90.5	100.7
Fumonisin (mg/kg)	<0.25	1.95	2.06	2.09
Deoxynivalenol (mg/kg)	<0.50	0.80	0.80	0.90
Grower (15–21 d)				
Aflatoxins (µg/kg)	<4.0	39.8	34.1	31.2
Zearalenone (µg/kg)	<25.0	149.9	145.5	151.5
T-2 toxin (µg/kg)	<25.0	87.8	94.0	91.4
Fumonisin (mg/kg)	<0.25	1.92	2.01	1.83
Deoxynivalenol (mg/kg)	<0.50	0.80	0.70	0.90
Finisher (22–42 d)				
Aflatoxins (µg/kg)	6.4	39.2	32.1	31.6
Zearalenone (µg/kg)	<25.0	166.6	158.2	157.3
T-2 toxin (µg/kg)	<25.0	93.9	101.7	93.0
Fumonisin (mg/kg)	0.50	2.32	2.16	2.17
Deoxynivalenol (mg/kg)	<0.50	0.70	0.90	0.80

^1^ CON, control group (basal diet); MMT, basal diet + multiple mycotoxins (25 µg/kg AFs, 135 µg/kg ZEA, 85 µg/kg T-2, 1.90 mg/kg FUM and 0.70 mg/kg DON); BSMD-1, basal diet + mixed mycotoxins + 1.0 g/kg broad-spectrum mycotoxin detoxifier-1 (UNIKE^®^ Plus, composed of bentonite, sepiolite, inactivated yeast and yeast extracts from Saccharomyces cerevisiae and botanicals); BSMD-2, basal diet + mixed mycotoxins + 1.5 g/kg broad-spectrum mycotoxin detoxifier-2 (TOXY-NIL^®^ Plus, composed of bentonite, sepiolite, inactivated yeast and yeast extracts, and botanicals).

**Table 2:** Ingredient composition of basal diet.

Ingredient (%)	Starter (1–14 d)	Grower (15–21 d)	Finisher (22–42 d)
Corn	57.10	61.37	63.64
Soybean meal (DH) (48% CP)	35.49	29.12	24.63
Full fat soybean	3.00	5.00	7.00
Soybean oil	0.02	0.51	1.33
Monocalcium phosphate	1.29	1.07	0.84
Limestone	1.16	1.06	0.95
Pellet binder	0.30	0.30	0.30
Salt	0.04	0.07	0.11
Broiler vit/min premix ^1^	0.20	0.20	0.20
DL-Methionine	0.32	0.28	0.24
L-Lysine HCl	0.22	0.22	0.15
L-Threonine	0.12	0.09	0.05
L-Valine	0.02	0.02	-
Sodium bicarbonate	0.36	0.32	0.25
Choline Chloride 60%	0.09	0.09	0.08
Antimould	0.20	0.20	0.20
Coccidiostat (Salinomycin)	0.05	0.05	-
Phytase (Quantum Blue 5G)	0.01	0.01	0.01
Total	100	100	100

^1^ Premix contains minerals and vitamins per kg premix, including Vitamin A 6,000,000 IU, Vitamin D3 1,200,000 IU, Vitamin E 30.00 g, Vitamin K 1.50 g, Vitamin B1 1.50 g, Vitamin B2 4.00 g, Vitamin B6 2.00 g, Vitamin B12 0.01 g, Niacin 25.00 g, Pantothenic acid 7.50 g, Biotin 0.20 g, Folic acid 1.00 g, Copper 7.50 g, Iron 20.00 g, Manganese 50.00 g, Zinc 50.00 g, Iodine 0.50 g, Selenium 0.15 g, Antioxidant 2.50 g, Filler (rice hull ground only) 554.29 g.

**Table 3:** Calculated nutrient composition for basal diet.

Nutrient (%)	Starter (1–14 d)	Grower (15–21 d)	Finisher (22–42 d)
Dry matter	87.67	87.67	87.70
ME for poultry (kcal/kg)	3000	3100	3200
Crude protein	23.00	21.50	19.50
Crude fat	3.26	4.20	5.41
Linoleic acid	1.57	2.05	2.68
Crude fiber	4.08	3.94	3.85
Ash	5.67	5.16	4.73
Digestible Lysine	1.28	1.15	1.02
Digestible Methionine	0.63	0.57	0.51
Digestible Cystine	0.32	0.30	0.29
Digestible Methionine + Cystine	0.95	0.87	0.80
Digestible Threonine	0.86	0.77	0.68
Digestible Tryptophan	0.27	0.24	0.22
Digestible Arginine	1.37	1.23	1.13
Digestible Valine	0.96	0.87	0.79
Digestible Isoleucine	0.87	0.78	0.72
Digestible Leucine	1.65	1.52	1.43
Calcium	0.96	0.87	0.78
Total Phosphorus	0.67	0.60	0.54
Available Phosphorus	0.48	0.44	0.39
Potassium	0.98	0.90	0.85
Choline (mg/kg)	1700	1600	1500
Sodium	0.16	0.16	0.16
Chloride	0.23	0.23	0.23

**Table 4:** Analyzed chemical composition of experimental diets.

Nutrient (%)	Dietary Treatments ^1^			
	CON	MMT	BSMD-1	BSMD-2
Starter (1–14 d)				
Dry matter	90.62	90.30	90.98	90.85
Protein	23.67	23.21	23.26	23.58
Fat	4.47	4.54	4.48	4.38
Fiber	2.02	2.07	2.00	2.04
Ash	5.69	5.61	5.62	5.61
Calcium	1.05	0.99	0.99	1.04
Total Phosphorus	0.67	0.68	0.67	0.68
Gross energy (kcal/kg)	4133.60	4129.48	4161.55	4158.96
Grower (15–21 d)				
Dry matter	92.28	90.66	92.84	90.32
Protein	21.69	21.97	22.02	22.52
Fat	5.50	5.59	5.35	5.10
Fiber	1.91	1.87	1.86	1.96
Ash	5.10	5.18	5.21	5.28
Calcium	0.89	0.91	0.86	0.88
Total Phosphorus	0.60	0.59	0.60	0.58
Gross energy (kcal/kg)	4218.51	4225.73	4232.41	4193.32
Finisher (22–42 d)				
Dry matter	90.78	91.27	90.64	91.42
Protein	20.23	20.82	20.55	20.95
Fat	6.43	6.51	6.53	6.76
Fiber	2.03	2.03	2.02	2.03
Ash	4.50	4.46	4.49	4.54
Calcium	0.80	0.79	0.77	0.79
Total Phosphorus	0.52	0.52	0.52	0.52
Gross energy (kcal/kg)	4217.14	4218.73	4211.88	4220.14

^1^ CON, control group (basal diet); MMT, basal diet + multiple mycotoxins (25 µg/kg AFs, 135 µg/kg ZEA, 85 µg/kg T-2, 1.90 mg/kg FUM and 0.70 mg/kg DON); BSMD-1, basal diet + mixed mycotoxins + 1.0 g/kg broad-spectrum mycotoxin detoxifier-1 (UNIKE^®^ Plus, composed of bentonite, sepiolite, inactivated yeast and yeast extracts from *Saccharomyces cerevisiae* and botanicals); BSMD-2, basal diet + mixed mycotoxins + 1.5 g/kg broad-spectrum mycotoxin detoxifier-2 (TOXY-NIL^®^ Plus, composed of bentonite, sepiolite, inactivated yeast, yeast extracts, and botanicals).

**Table 5:** Growth performance of broilers fed diets contaminated with mixed mycotoxins and supplemented with broad-spectrum mycotoxin detoxifiers.

Parameter	Dietary Treatment ^1^				SEM	*p* Value
	CON	MMT	BSMD-1	BSMD-2		
IBW (g)	44.98	45.08	45.10	44.97	0.13	0.9790
1–14 d						
FBW (g)	541.00	531.11	538.32	538.40	1.73	0.213
BWG (g)	496.02	486.03	493.22	493.43	1.71	0.195
FI (g)	572.81	566.52	569.50	570.57	2.61	0.873
FCR	1.16	1.17	1.16	1.16	0.004	0.784
15–21 d						
FBW (g)	1118.54	1098.82	1110.07	1109.82	4.51	0.511
BWG (g)	577.54	567.71	571.75	571.42	3.60	0.828
FI (g)	798.98	825.49	824.74	825.55	5.06	0.166
FCR	1.38	1.45	1.44	1.45	0.01	0.173
22–35 d						
FBW (g)	2426.93	2375.17	2413.78	2407.41	20.08	0.840
BWG (g)	1308.39	1276.34	1303.71	1297.59	17.08	0.924
FI (g)	2194.63	2176.85	2206.40	2196.62	15.27	0.930
FCR	1.68	1.71	1.69	1.69	0.01	0.888
36–42 d						
FBW (g)	3025.18	2937.47	2979.08	2979.39	45.90	0.935
BWG (g)	598.25	562.30	565.30	571.98	31.51	0.980
FI (g)	1250.89	1238.91	1154.95	1214.52	22.43	0.455
FCR	2.09	2.20	2.04	2.12	0.14	0.871
1–42 d						
FBW (g)	3025.18	2937.47	2979.08	2979.39	45.90	0.935
BWG (g)	2980.20	2892.39	2933.99	2934.42	45.93	0.935
FI (g)	4817.32	4807.77	4755.59	4807.25	34.93	0.931
FCR	1.62	1.66	1.62	1.64	0.02	0.729

^1^ CON, control group (basal diet); MMT, basal diet + multiple mycotoxins (25 µg/kg AFs, 135 µg/kg ZEA, 85 µg/kg T-2, 1.90 mg/kg FUM and 0.70 mg/kg DON); BSMD-1, basal diet + mixed mycotoxins + 1.0 g/kg broad-spectrum mycotoxin detoxifier-1 (UNIKE^®^ Plus, composed of bentonite, sepiolite, inactivated yeast and yeast extracts from *Saccharomyces cerevisiae* and botanicals); BSMD-2, basal diet + mixed mycotoxins + 1.5 g/kg broad-spectrum mycotoxin detoxifier-2 (TOXY-NIL^®^ Plus, composed of bentonite, sepiolite, inactivated yeast, yeast extracts, and botanicals). SEM, standard error of the mean; IBW, initial body weight; FBW, final body weight; BWG, body weight gain; FI, feed intake; FCR, feed conversion ratio.

**Table 6:** Antibody titers to Newcastle disease and Infectious bronchitis of broiler chickens fed diets contaminated with mixed mycotoxins and supplemented with broad-spectrum mycotoxin detoxifiers.

Parameter	Dietary Treatment ^1^				SEM	*p* Value
	CON	MMT	BSMD-1	BSMD-2		
ND titer (log_2_)	2.45	2.39	2.44	2.48	0.02	0.646
IB titer (log_2_)	2.87	2.82	2.90	2.93	0.03	0.661

^1^ CON, control group (basal diet); MMT, basal diet + multiple mycotoxins (25 µg/kg AFs, 135 µg/kg ZEA, 85 µg/kg T-2, 1.90 mg/kg FUM and 0.70 mg/kg DON); BSMD-1, basal diet + mixed mycotoxins + 1.0 g/kg broad-spectrum mycotoxin detoxifier-1 (UNIKE^®^ Plus, composed of bentonite, sepiolite, inactivated yeast and yeast extracts from Saccharomyces cerevisiae and botanicals); BSMD-2, basal diet + mixed mycotoxins + 1.5 g/kg broad-spectrum mycotoxin detoxifier-2 (TOXY-NIL^®^ Plus, composed of bentonite, sepiolite, inactivated yeast, yeast extracts, and botanicals). SEM, standard error of the mean; ND, Newcastle disease; IB, Infectious bronchitis.

**Table 7:** Oxidative stress and inflammation indices of broiler chickens fed diets contaminated with mixed mycotoxins and supplemented with broad-spectrum mycotoxin detoxifiers.

Parameter	Dietary Treatment ^1^				SEM	*p* Value
	CON	MMT	BSMD-1	BSMD-2		
GSH-Px (ng/mL)	13.20	12.07	12.78	12.72	0.02	0.983
MDA (µM/100 µL)	8.34	8.65	8.54	8.64	0.05	0.942
IL-6 (pg/mL)	98.35	122.03	109.19	109.12	0.03	0.903
IL-10 (pg/mL)	4.07 ^b^	3.58 ^b^	4.24 ^a^	4.26 ^a^	0.01	0.015
AST (U/L)	506.00	519.63	513.07	511.71	0.01	0.940
ALT (U/L)	3.63	4.13	3.83	4.00	0.01	0.638
GGT (U/L)	22.21	23.71	22.33	23.33	0.01	0.777

^a,b^ Means within the same row with different superscripts differ significantly (*p* < 0.05). ^1^ CON, control group (basal diet); MMT, basal diet + multiple mycotoxins (25 µg/kg AFs, 135 µg/kg ZEA, 85 µg/kg T-2, 1.90 mg/kg FUM and 0.70 mg/kg DON); BSMD-1, basal diet + mixed mycotoxins + 1.0 g/kg broad-spectrum mycotoxin detoxifier-1 (UNIKE^®^ Plus, composed of bentonite, sepiolite, inactivated yeast and yeast extracts from *Saccharomyces cerevisiae* and botanicals); BSMD-2, basal diet + mixed mycotoxins + 1.5 g/kg broad-spectrum mycotoxin detoxifier-2 (TOXY-NIL^®^ Plus, composed of bentonite, sepiolite, inactivated yeast, yeast extracts, and botanicals). SEM, standard error of the mean; GSH-Px, glutathione peroxidase; MDA, malondialdehyde; IL-6, interleukin 6; IL-10, interleukin 10; AST, aspartate aminotransferase; ALT, alanine aminotransferase; GGT, gamma glutamyl transferase.

**Table 8:** Jejunal morphology of broiler chickens fed diets contaminated with mixed mycotoxins and supplemented with broad-spectrum mycotoxin detoxifiers.

Parameter	Dietary Treatment ^1^				SEM	*p* Value
	CON	MMT	BSMD-1	BSMD-2		
Villus height (µm)	1230.81 ^a^	1084.51 ^b^	1219.17 ^a^	1224.97 ^a^	21.05	0.043
Villus width (µm)	172.17	160.38	167.91	169.37	2.48	0.436
Surface area (µm^2^ × 10^−3^)	665.41 ^a^	546.15 ^b^	642.75 ^a^	651.51 ^a^	15.38	0.028
Crypt depth	201.86	229.92	212.68	213.65	5.30	0.361
Villus height: Crypt depth	6.098 ^a^	4.718 ^b^	5.733 ^a^	5.732 ^a^	0.15	0.007

^a,b^ Means within the same row with different superscripts differ significantly (*p* < 0.05). ^1^ CON, control group (basal diet); MMT, basal diet + multiple mycotoxins (25 µg/kg AFs, 135 µg/kg ZEA, 85 µg/kg T-2, 1.90 mg/kg FUM and 0.70 mg/kg DON); BSMD-1, basal diet + mixed mycotoxins + 1.0 g/kg broad-spectrum mycotoxin detoxifier-1 (UNIKE^®^ Plus, composed of bentonite, sepiolite, inactivated yeast and yeast extracts from *Saccharomyces cerevisiae* and botanicals); BSMD-2, basal diet + mixed mycotoxins + 1.5 g/kg broad-spectrum mycotoxin detoxifier-2 (TOXY-NIL^®^ Plus, composed of bentonite, sepiolite, inactivated yeast, yeast extracts, and botanicals). SEM, standard error of the mean.

**Table 9:** Fatty liver scores of broiler chickens fed diets contaminated with mixed mycotoxins and supplemented with broad-spectrum mycotoxin detoxifiers.

Parameter	Dietary Treatment ^1^				SEM	*p* Value
	CON	MMT	BSMD-1	BSMD-2		
Prevalence of Fatty Liver Score ^2^ (%)						
Score 1	87.50	37.50	56.25	56.25	0.14	0.212
Score 2	12.50	50.00	37.50	43.75	0.16	0.196
Score 3	0.00	12.50	6.25	0.00	0.09	0.278
Score 4	0.00	0.00	0.00	0.00	-	-
Average score	1.13 ^b^	1.75 ^a^	1.50 ^ab^	1.44 ^ab^	0.08	0.031

^a,b^ Means within the same row with different superscripts differ significantly (*p* < 0.05). ^1^ CON, control group (basal diet); MMT, basal diet + multiple mycotoxins (25 µg/kg AFs, 135 µg/kg ZEA, 85 µg/kg T-2, 1.90 mg/kg FUM and 0.70 mg/kg DON); BSMD-1, basal diet + mixed mycotoxins + 1.0 g/kg broad-spectrum mycotoxin detoxifier-1 (UNIKE^®^ Plus, composed of bentonite, sepiolite, inactivated yeast and yeast extracts from *Saccharomyces cerevisiae* and botanicals); BSMD-2, basal diet + mixed mycotoxins + 1.5 g/kg broad-spectrum mycotoxin detoxifier-2 (TOXY-NIL^®^ Plus, composed of bentonite, sepiolite, inactivated yeast, yeast extracts, and botanicals). SEM, standard error of the mean ^2^ Fatty liver score: Score 1, normal liver with a dark red color; Score 2, mild fatty liver hemorrhagic syndrome (FLHS), characterized by a slightly yellow liver and mild hemorrhages; Score 3, moderate FLHS, presenting as a light yellowish-red liver with moderate hemorrhages; Score 4, severe FLHS, characterized by an enlarged, putty-colored liver with large or massive hemorrhages.

**Table 10:** Liver lesion score in broiler chickens fed diets contaminated with mixed mycotoxins and supplemented with broad-spectrum mycotoxin detoxifiers.

Parameter	Dietary Treatment ^1^				SEM	*p* Value
	CON	MMT	BSMD-1	BSMD-2		
Prevalence of Liver Lesion Score ^2^ (%)						
Score 0	12.50	0.00	12.50	0.00	0.09	0.316
Score 1	31.25	37.50	31.25	25.00	0.16	0.843
Score 2	50.00	56.25	43.75	31.25	0.17	0.577
Score 3	6.25	6.25	12.50	43.75	0.14	0.198
Score 4	0.00	0.00	0.00	0.00	-	-
Average score	1.500	1.688	1.563	2.188	0.12	0.171

^1^ CON, control group (basal diet); MMT, basal diet + multiple mycotoxins (25 µg/kg AFs, 135 µg/kg ZEA, 85 µg/kg T-2, 1.90 mg/kg FUM and 0.70 mg/kg DON); BSMD-1, basal diet + mixed mycotoxins + 1.0 g/kg broad-spectrum mycotoxin detoxifier-1 (UNIKE^®^ Plus, composed of bentonite, sepiolite, inactivated yeast and yeast extracts from *Saccharomyces cerevisiae* and botanicals); BSMD-2, basal diet + mixed mycotoxins + 1.5 g/kg broad-spectrum mycotoxin detoxifier-2 (TOXY-NIL^®^ Plus, composed of bentonite, sepiolite, inactivated yeast, yeast extracts, and botanicals). SEM, standard error of the mean. ^2^ Liver lesion score: Score 0, normal liver; Score 1, mild fatty degeneration, characterized by vacuolar (hydropic) degeneration of hepatic cells affecting <50% of the tissue area; Score 2, extensive vacuolar degeneration throughout the hepatic parenchyma with occasional foci of hepatitis; Score 3, severe vacuolar degeneration of the hepatic parenchyma with moderate to severe hepatitis, characterized by infiltration of inflammatory cells into the parenchyma; and Score 4, severe, extensive hepatitis with fibrosis and features suggestive of possible cirrhosis, including bile duct proliferation and hyperplasia.

## Data Availability

The original contributions presented in this study are included in the article. Further inquiries can be directed to the corresponding author.

## Abbreviations

The following abbreviations are used in this manuscript:

BSMD	Broad-spectrum mycotoxin detoxifiers
MMT	Multiple mycotoxin
AF	Aflatoxin
FUM	Fumonisin
ZEA	Zearalenone
DON	Deoxynivalenol
MDA	Malondialdehyde
CD	Crypt depth
VW	Villus width
VH	Villus height
ND	Newcastle disease
IB	Infectious bronchitis
